# Lingual orthodontic treatment duration: performance of two different completely customized multi-bracket appliances (Incognito and WIN) in groups with different treatment complexities

**DOI:** 10.1186/1746-160X-10-46

**Published:** 2014-11-01

**Authors:** Michael Knösel, Elisabeth Klang, Hans-Joachim Helms, Dirk Wiechmann

**Affiliations:** Department of Orthodontics, University Medical Center Göttingen (UMG), 37099 Göttingen, Germany; Orthodontic Practice, Lindenstrasse 44, 49152 Bad Essen, Germany; Department of Medical Statistics, University Medical Center Göttingen (UMG), 37099 Göttingen, Germany; Department of Orthodontics, Hannover Medical School (MHH), 30625 Hannover, Germany

**Keywords:** Orthodontic treatment duration, Lingual multibracket appliance, Incognito appliance, WIN appliance, In vivo

## Abstract

**Introduction:**

The occurrence of side-effects of fixed orthodontic therapy, such as white-spot lesions and root resorption, are known to be significantly more frequent with increasing duration of treatment. Multi-bracket treatment should be as short as possible, in order to minimize the risks of collateral damage to teeth. The aim of this non-randomized clinical trial was to compare treatment duration with each of two types of customized lingual orthodontic appliances (Incognito, 3 M-Unitek; WIN, DW LingualSystems), taking into account treatment complexity. The null-hypothesis was that there would be no significant difference in active orthodontic treatment duration between them.

**Methods:**

Of 402 potentially eligible participants, a population sample of n = 376 subjects (n_Incognito_ = 220; n_WIN_ = 156; m/f 172/204; mean age ± SD 17.3 ± 7.7Y) treated in one orthodontic center (Bad Essen, Germany) with completely customized lingual appliances in upper and lower permanent dental arches was recruited with the inclusion criterion of initiated and completed lingual multi-bracket treatment within the assessment period of April 1st 2010 – Nov 30, 2013, and the exclusion criterion of less than 24 bracketed teeth. We used four-factorial ANOVA to assess the impact of the following factors: initial degree of severity of malocclusion (mild to moderate, S1; severe, S2), appliance type (Incognito; WIN), sex, and age group (<=16; >16 Y) on the duration of lingual multi-bracket treatment.

**Results:**

Overall, mean treatment duration was 21.7 (SD 7.2) months, which was significantly shorter for WIN for both sub-groups of treatment complexity (S1: 17.96 mo; S2: 20.49 mo) compared to Incognito (S1: 22.7 mo; S2: 29.79 mo). ANOVA revealed a significant influence of the main effects ‘appliance type’, and ‘severity’, independent of each other. Therefore, the null-hypothesis was rejected.

**Conclusion:**

In terms of treatment duration, the WIN appliance performed significantly better than the Incognito appliance. Consequently, subjects treated with the WIN appliance are expected to be exposed to lower risks of the typical side-effects associated with longer multi-bracket treatment durations, such as root resorption and enamel decalcification.

## Introduction

Multi-bracket treatment (MB) is considered to be the most rational orthodontic treatment approach, as it enables the correction of tooth position three-dimensionally, with a minimum or often even no need for patient compliance in order to achieve good occlusions. Nonetheless, patient compliance is needed to avoid the problem of enamel decalcification and incipient caries. Inadequate oral hygiene during MB is known to promote the formation of white-spot lesions (WSLs). However, also a certain percentage of subjects achieving an adequate standard of daily oral hygiene are prone to develop enamel decalcifications with increasing treatment duration. In addition to the occurrence of WSLs, apical external root resorption is a much feared side-effect of fixed orthodontic therapy which, based on meta-analyses, is well known to increase significantly in both severity and frequency, the longer MB treatment duration is [1–3]. Therefore, multi-bracket treatment should be as short as possible, to minimize the risks of collateral damage to teeth. Accordingly, self-ligating bracket systems have often been proposed to accelerate treatment and advance de-bonding. However, whilst individual studies have reported minor differences in treatment duration in favor of self-ligating appliances [4, 5], at the level of RCTs or systematic reviews, there has only been limited or no evidence that the self-ligation technique might shorten tooth alignment or MB treatment duration [6, 7].

From the perspective of reducing frequencies of enamel decalcification, the lingual bracket approach seems favorable [8], and reasons may be seen in enhanced saliva wetting and self-cleansing of enamel surfaces [9]. Moreover, considered as an esthetic benefit, lingual WSLs do not impair dentofacial appearance, which is otherwise a very frequent problem in finished cases treated with conventional labial-side MB. So, in order to minimize both the side-effects of WSLs and root-resorption, it might be preferable to choose a lingual appliance with a high degree of efficiency for reducing treatment times and, associated with this, also the side-effect of root resorption.

**Figure 1:**
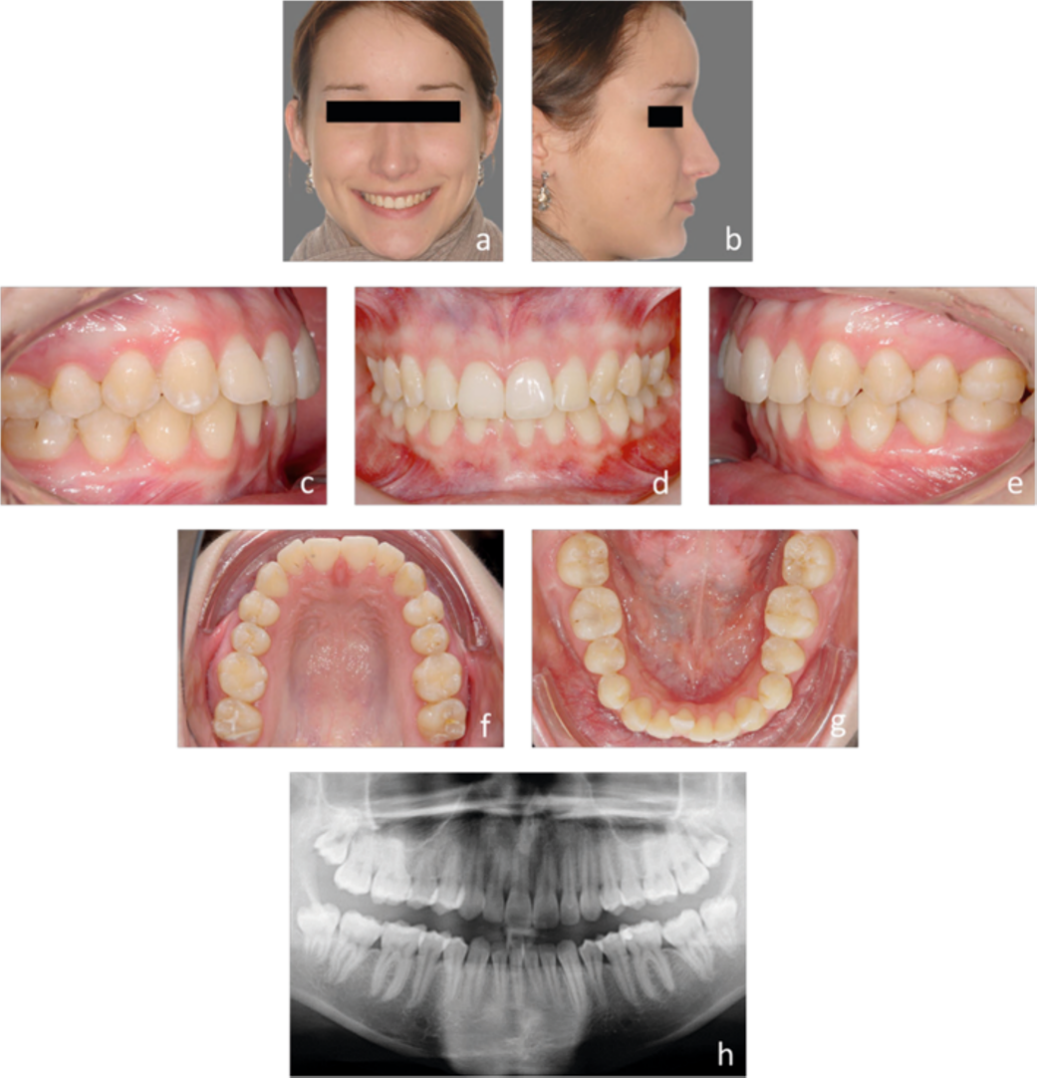
**a-h: Representative initial situation of an S1 treatment complexity.** Juvenile patient with moderate frontal crowding in both arches and a deep bite (S1).

**Figure 2:**
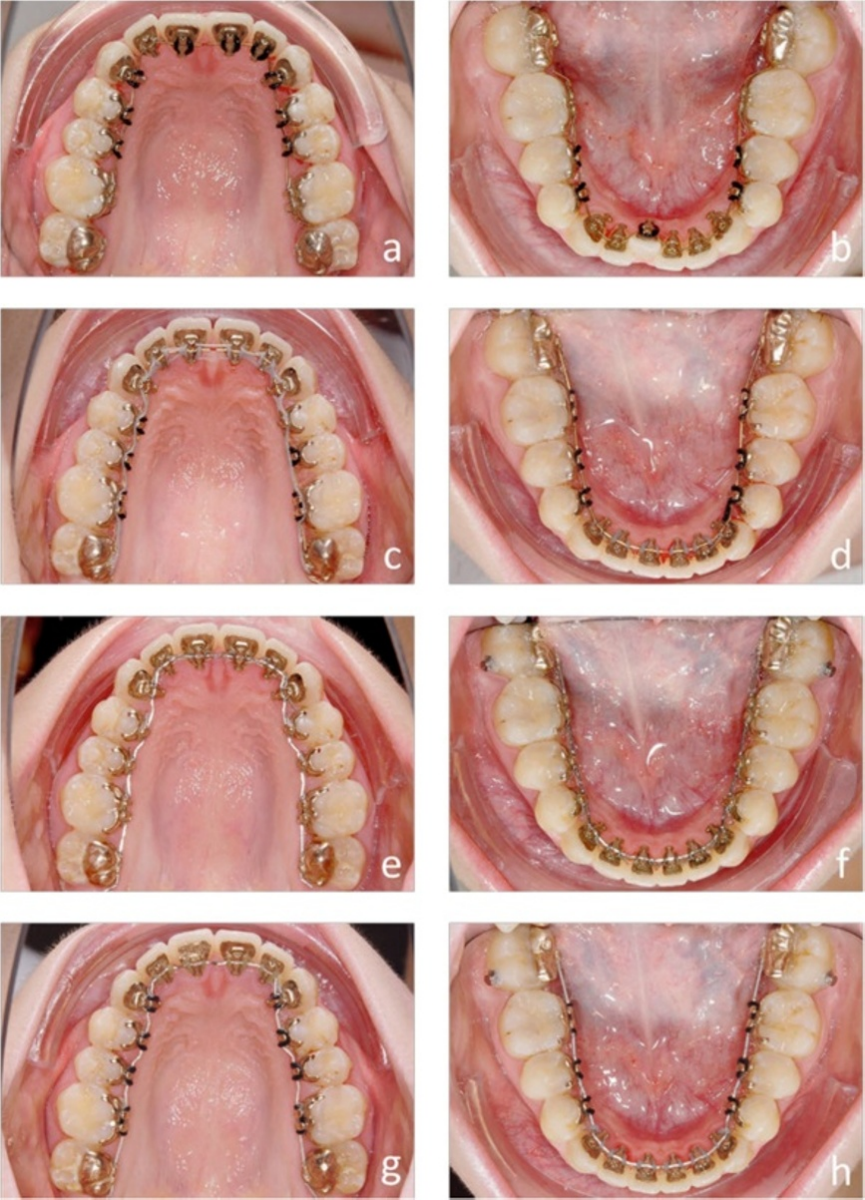
**a-h Progress of the lingual orthodontic treatment with the Incognito appliance.** Despite the lower frontal crowding all brackets could be bonded right from the beginning of treatment **(a and b)**. Complete derotation is accomplished by rectangular SE-Niti archwires **(c and d)**. SS archwires are inserted for class II correction with inter-maxillary elastics mainly on the left side. Labial metal buttons are bonded on the labial side of the lower second molars to facilitate the insertion of the elastics **(e and f)**. Final TMA archwires are used for finishing. A manual finishing bend was necessary to correct the tip problem of the upper right central incisor **(g and h)**.

**Figure 3:**
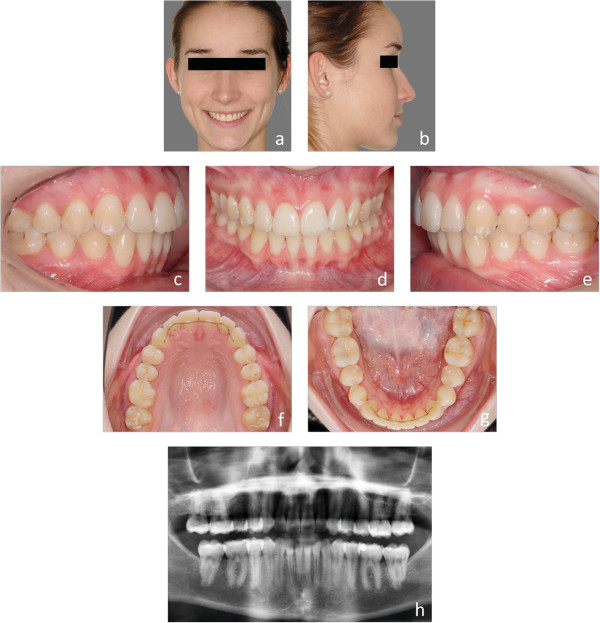
**a-h: Final result after 22 month of lingual orthodontic treatment.** Bonded retainers are used for retention in both arches **(f and g)**.

**Figure 4:**
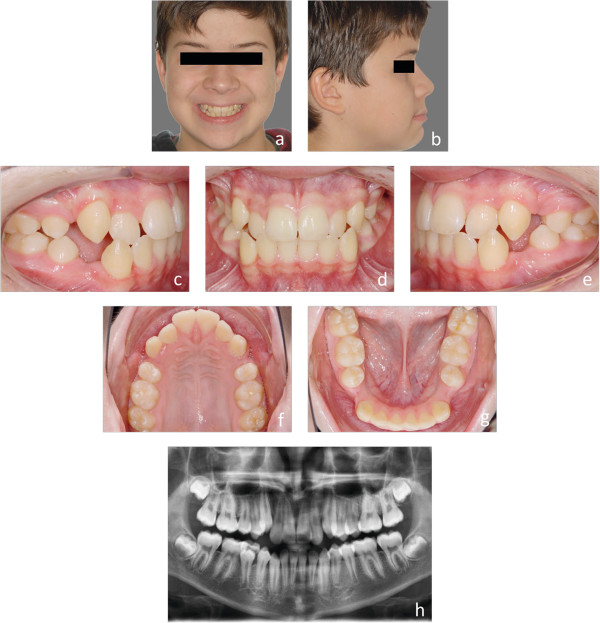
**a-h: Representative initial situation of an S2 treatment complexity.** Juvenile patient with a class III tendency. Because of the frontal crowding **(h)** the four first premolars were extracted (S2).

**Figure 5:**
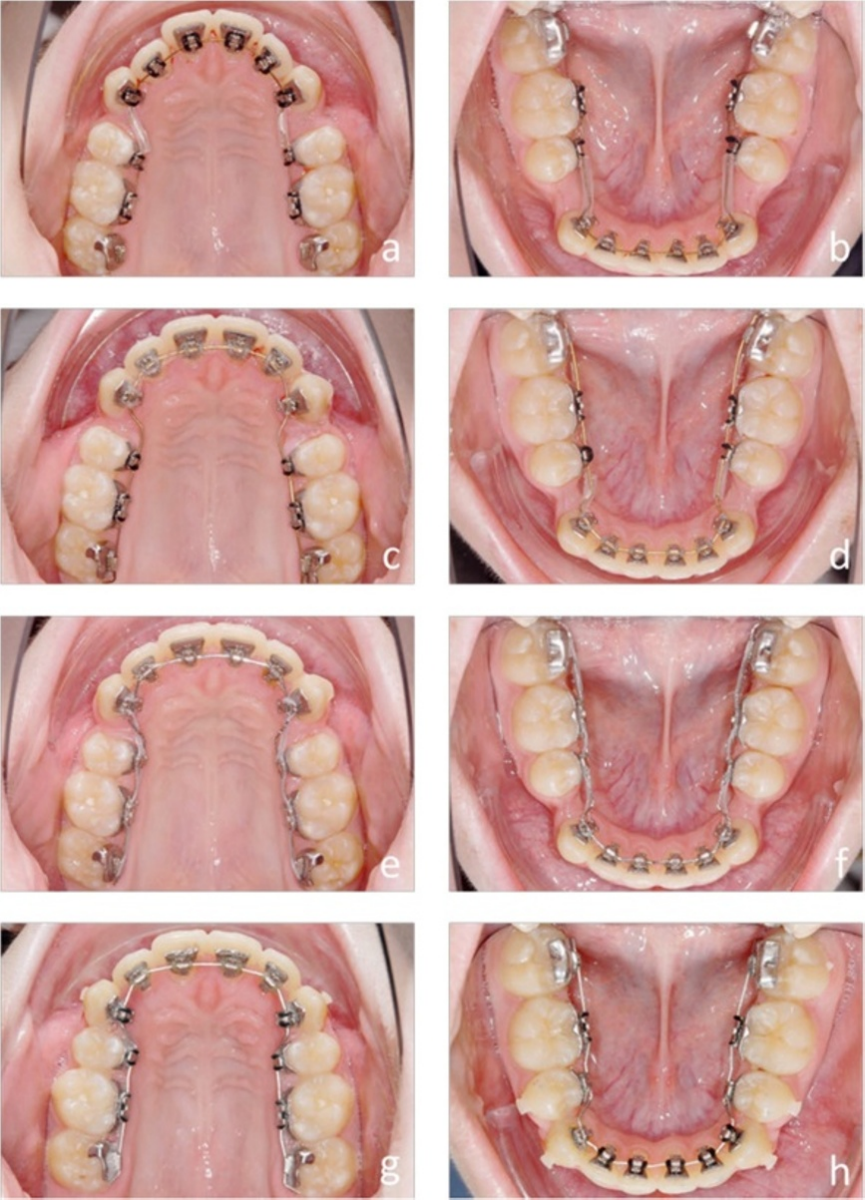
**a-h: Progress of the lingual orthodontic treatment with the WIN appliance.** Round SE-Niti archwires are inserted directly after indirect bonding **(a and b)**. The rectangular SE-Niti archwires are straight in the lateral segments to prepare for space closure **(c and d)**. The residual spaces are closed on the SS archwires with the help of power chains **(e and f)**. For final torque control 0.018”x0.018” TMA archwires are used **(g and h)**. The TMA archwires are individual in the lateral segments. No supplementary finishing bends were necessary in both arches.

**Figure 6:**
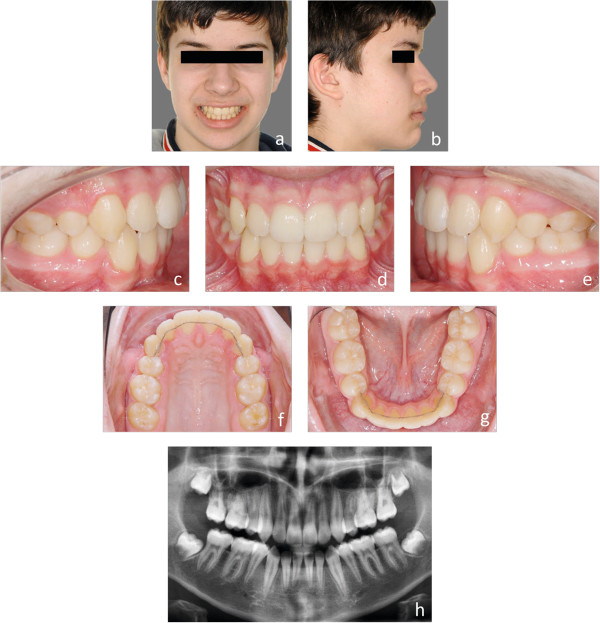
**a-h: Final result after 17 month of lingual orthodontic treatment.** The parallelism of the roots demonstrate a good tip control during space closure **(c, d, e and h)**. Bonded retainers from 5-5 are used for retention in both arches **(f and g)**.

Customized lingual bracket systems with individual base contours have been reported to be superior to ready-made brackets or half-customized bracket systems, in terms of fitting and quality of treatment results [10]. Therefore, we compared the first generation of a completely customized lingual appliance (Incognito, 3 M-Unitek, Top-Service für Lingualtechnik, Bad Essen, Germany) [10], with the one from the subsequent generation (WIN, DW LingualSystems, Bad Essen, Germany) (Figures 1a-h, 2a-h, 3a-h, 4a-h, 5a-h and 6a-h).

Typical features known to slow down the speed of fixed orthodontic treatment have been summarized in systematic reviews [6], including extraction treatments compared to non-extraction variants (with a significant positive correlation between numbers of extracted teeth and increased treatment duration), presence of impacted canines, severity of the initial malocclusion (bearing in mind that there is little evidence regarding differences in treatment duration between the various types of malocclusions), and, to an unknown extent, the level of skill and number of operators involved, and patients’ compliance [11–13]. Apart from these factors, differences in subjects’ ages were not found to be significantly correlated with treatment duration, provided that patients were in possession of permanent dentition [6].

### Aim of the study

The aim of this study was to compare the speed of treatment with either of two types of customized lingual orthodontic appliances (Incognito, 3 M-Unitek; WIN, DW LingualSystems), taking into account factors known to have a potential influence on treatment duration, such as the degree of complexity of treatment and severity of malocclusion, and the patient-related factors age group (<=16Y/>16Y) and gender.

The null-hypothesis was that there would be no significant difference in active orthodontic treatment duration between either of two completely customized lingual appliances (Incognito; WIN).

## Methods

Before the start of this single-center, non-randomized clinical trial on the duration of fixed orthodontic treatment with two lingual multi-bracket appliances, approval was obtained from the Medical University of Hannover (Germany) Ethics Committee (#1189/2011), and all patients or their guardians were required to give informed consent prior to commencement of the trial.

Of 402 potentially eligible participants, a population of n = 376 (n_Incognito_ = 220 subjects/5,696 bracketed teeth; n_WIN_ = 156 subjects/4,096 bracketed teeth; n_males/females_ = 172/204; mean age ± SD 17.3 ± 7.7Y) subjects treated in one orthodontic center (Bad Essen, Germany), under the supervision of the same operator (DW), with completely customized lingual appliances in the upper and lower permanent dental arches was recruited, adopting the inclusion criterion of initiated and completed lingual multi-bracket treatment within the assessment period April 1st 2010 – Nov 30, 2013, and the exclusion criterion of less than 24 teeth bracketed. Archwire sequence typically started with 0.012” or 0.014” SE-Niti, followed by 0.016” × 0.022” SE-Niti, and 0.016” × 0.024” SS, and complemented by 0.018” × 0.018” TMA archwires based on individual clinical situations and case requirements, but using the same clinical guidelines and rationale for both appliance types. Extraction and Herbst treatments comprised additional 0.018” × 0.025” SE-Niti and 0.018” × 0.025” SS archwires. In order to meet individual case requirements, all TMA and SS archwires were completely customized using a bending robot. Records included the subjects’ ages, gender, type of appliance (Incognito; WIN), time points of bracketing (T0), and de-bracketing (T1), information about the initial orthodontic situation and auxiliary appliances used.

### Classification of treatment complexities

Individual treatment complexities were classified using grouped severity of malocclusion of either mild to moderate (S1), or severe malocclusions and difficult (S2) treatments. S1/S2 discrimination criteria included extractions (thereby including subjects with severe crowding, without the need to perform error-prone, tooth-space analyses); agenesis of at least one tooth (space opening or gap-closing treatment), impacted or dislocated teeth, use of temporary anchorage devices (TADs), Herbst appliances, and orthognathic surgery. Examples of subjects allocated to groups S1 and S2 are depicted by Figures 1a-h (S1), and 4a-h (S2).

Table 1 provides detailed information about the subjects, as well as the period of time between bracketing and de-bonding. A note was made of the number of brackets lost and re-bonded during treatment (Table 1).

**Table 1:** **Descriptive analysis of subjects’ ages, gender, bracket losses, and treatment duration**

	Incognito (n = 220)	WIN (n = 156)	Total (n = 376)
Patients’ ages at T0 [years; Mean ± SD]	17.27 ± 7.20	17.42 ± 8.45	17.33 ± 7.73
Treatment Duration [months; Mean ± SD]	24.27 ± 7.4	18.12 ± 5.11	21.72 ± 7.21
Sex [m/f] n (%)	99/121 (45.0%/55.0%)	73/83 (46.8%/53.2%)	172/204 (45.7%/54.3%)
Brackets lost per subject with re-bonding Mean [Min/Max/Median]	2.54 [0/12/2]	2.15 [0/16/2]	2.38 [0/16/2]
Brackets lost per subject w/o re-bonding Mean [Min/Max/Median]	0.5 [0/5/0]	0.44 [0/4/0]	0.47 [0/5/0]

### Statistical analysis

Subject- and treatment-specific features, such as treatment time, bracket losses, and age-distribution in the various groups of different treatment complexities were descriptively analyzed (means and standard deviations). A four-factorial ANOVA was used to assess the impact of the factors of initial degree of severity of malocclusion (mild to moderate, S1; severe, S2), appliance type (Incognito; WIN), sex (m; f) and subjects’ ages (grouped < = 16; >16 Y) on the duration of lingual multi-bracket treatment. The significance level was set at α = 5%. The statistical software packages SAS 9.3 (SAS Institute; Cary, NC, USA) and STATISTICA 10 (StatSoft. Inc.; Tulsa, OK, USA) were used for the statistical analyses.

## Results

**Figure 7:**
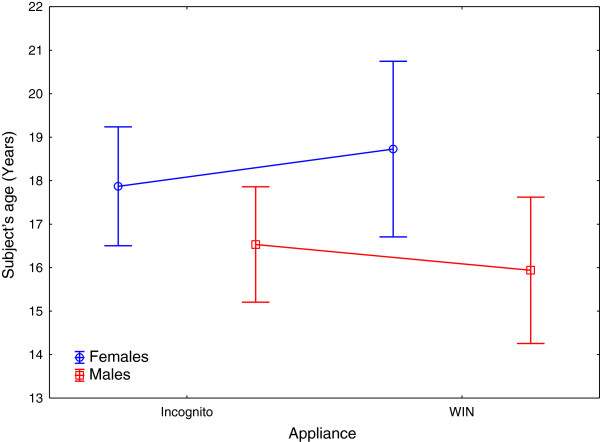
**Distribution of subjects’ ages at the start of treatment separated by appliance type and sex:** ***T*** **-Test revealed no significant difference in terms of age of subjects between the two appliance groups (WIN, Incognito) (mean/SD Incognito: 17.3 Y/7.2; WIN 17.4 Y/8.4; p = 0.85).**

**Figure 8:**
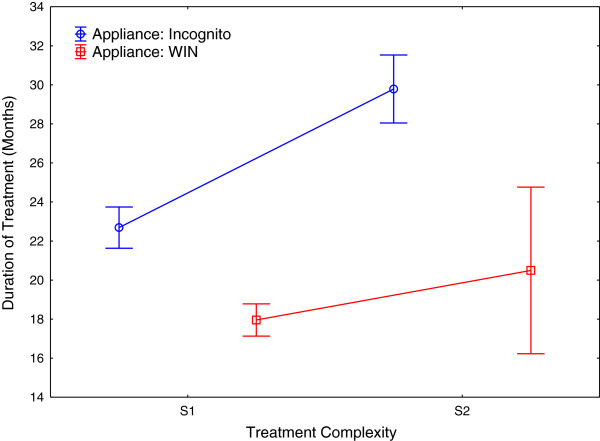
**Impact of degrees of treatment complexities and severities of malocclusion on treatment duration with the two types of appliances.**

**Figure 9:**
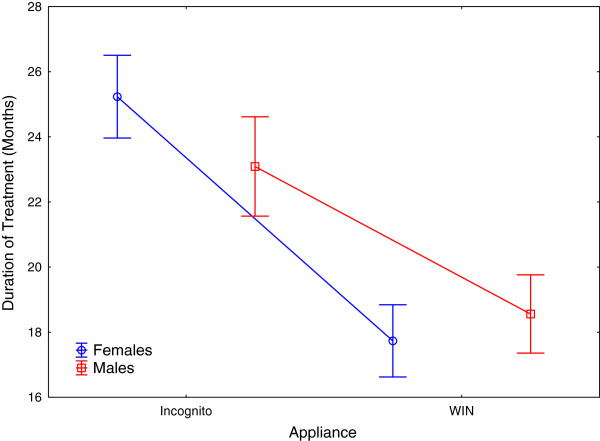
**Treatment duration plotted separately for appliance type and gender.**

**Figure 10:**
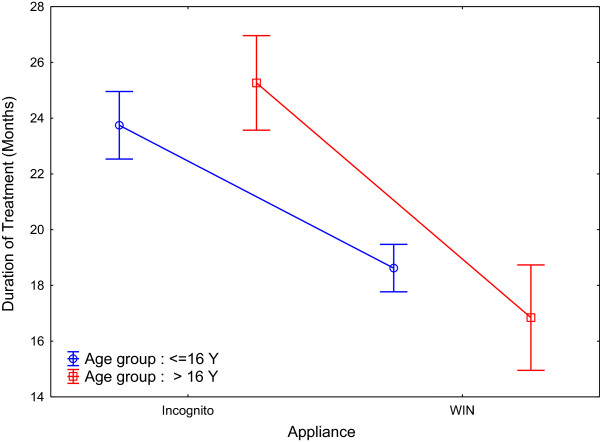
**Treatment duration plotted separately for appliance type and age group.**

Overall, mean lingual orthodontic treatment duration was 21.7 (SD 7.2) months, which was significantly shorter in patients treated with the WIN appliance, for both sub-groups of treatment complexities (S1: 17.96 mo, p < 0.0001; S2: 20.49 mo, p = 0.0006) compared to Incognito (S1: 22.7 mo; S2: 29.79 mo; Tables 1 and 2; Figure 7). ANOVA revealed a significant influence of the main effects ‘appliance type’, and ‘severity’, not dependent on each other (Tables 3, 4 and 5; Figures 8, 9 and 10). Using the Pearson Chi-square test, no significant difference was found in terms of frequencies of bracket losses (p = 0.24, Table 1).

**Table 2:** **Duration of treatment [Months] in sub-groups of severities of malocclusion 1 (mild to moderate cases) and 2 (severe cases)**

Severity	Appliance	N	Means	SD	Min	Max	Median
S1	Incognito	171	22.69	6.99	3.26	41.89	22.14
S1	WIN	146	17.96	5.03	3.97	29.23	17.98
S2	Incognito	49	29.79	6.06	16.01	42.86	30.62
S2	WIN	10	20.49	5.96	11.97	28.97	19.53
All Groups		376	21.72	7.21	3.26	42.88	20.94

The differences between WIN and Incognito were highly significant for both S1 (p < 0.0001) and S2 (p = 0.0006).

**Table 3:** ***T*** **-Test to detect differences in treatment time by degree of treatment complexity, but with no breakdown by appliance type**

	Mean (SD) - S1	Mean (SD) - S2	p - 2-sided	N - S1	N - S2
**Treatment duration [Months]**	20.51 (6.59)	28.21 (6.95)	<0.0001	317	59

Treatment duration was significantly longer in subjects classified as having a ‘severe’ type of malocclusion.

**Table 4:** **Chi-square-test was used to check whether the distribution of treatment complexities judged as ‘severe’ were equally distributed among the subjects treated with either of the two appliance types**

	Appliance	Severity - 1	Severity - 2	Row - totals
**Count**	Incognito	171 (77.73%)	49 (22.27%)	220
**Count**	WIN	146 (93.59%)	10 (6.41%)	156
**Count**	All Groups	317 (84.30)	59 (15.69)	376

As we detected a more significant difference (p < 0.0001), the factor of grouped severity of malocclusion was included in the ANOVA as a main factor.

**Table 5:** **Treatment duration by complexity of malocclusion and appliance type**

Effect	p-value
**Severity of Malocclusion**	<.0001
**Appliance Type**	<.0001
Severity Maloccl* Appliance	0.13
**Sex**	0.73
Severity Maloccl * Sex	0.78
Appliance * Sex	0.44
Severity Maloccl* Appliance * Sex	0.63
**Age (grouped < = / >16Y)**	0.81
Severity Maloccl * Age	0.75
Appliance * Age	0.85
Severity Maloccl * Appliance *Age	0.33
Sex * Age	0.51
Severity Maloccl * Sex * Age	0.64
Appliance * Sex * Age	0.61
Severity Maloccl * Appliance * Sex * Age	0.75

*denotes interactions between factors.

Results of the four-factorial ANOVA which was used to investigate the impact of the factors treatment complexity/severity of malocclusion (S1, S2), appliance type (Incognito/WIN), sex (f = 0,m = 1), and age group (0 = <=16, 1= >16 years).

There was a significant influence of severity of malocclusion and type of appliance, but no other significant effect or interaction between any investigated factors.

Consequently, the null-hypothesis of no significant difference in active orthodontic treatment duration between either of two customized lingual orthodontic appliances (Incognito, 3 M-Unitek; WIN, DW LingualSystems) was rejected.

## Discussion

Considering the abundance of reports available regarding mean treatment times for conventional labial multi-bracket treatment, investigations of treatment durations of fixed lingual treatment have been few and far between [6, 14–16]. The factors of extraction therapy, impacted canines and deciduous teeth have been identified as factors having a significant influence on treatment times based on systematic reviews [6, 16, 17], while other authors have also reported the presence of severe maxillary crowding or large over-jets being associated with long treatment times [17, 18]. In order to address these factors having a potential influence and to separate the effects of appliance type and treatment complexity, we created two groups of malocclusion severities, where the group of ‘difficult’ cases (S2) comprised most situations known to cause delays in treatment time. We allocated subjects requiring gap closures after extraction of teeth to group S2, and the subset of extraction cases also covered all subjects with severe crowding, however, without the need for error-prone, dental arch-space analyses. The parameter ‘extraction’ also covers all cases with large overjets that were not treated by fixed functional appliances, which were also an S2 inclusion criterion. In addition, space opening or gap closing treatment in cases of agenesis of at least one tooth and impacted or dislocated teeth were allocated to group S2. Subjects requiring specific additional appliances, such as TADs or auxiliary Herbst appliances, or requiring complex and time-consuming interdisciplinary logistics by orthognathic surgery were also subsumed under category S2.

As there is only little evidence that further differences between malocclusions significantly affect mean treatment times, there was no additional sub-analysis based on more detailed occlusal features [6].

### Standardization of the treatment routine

The skill and number of operators involved in the treatment of orthodontic patients have been identified as factors that may potentially influence treatment duration [6], as are the individual time spent by clinicians for finishing and detailing [19]. In order to minimize and standardize these treatment-related factors in this trial, the indirect bonding procedure was performed in one orthodontic center, and was identical for both types of appliances, which was ensured by implementing a rigid protocol [20]. Individual archwires were manufactured using bending robots, for maximum precision and reproducability of archwire forms and bending.

### Treatment time for lingual compared to buccal

Mean treatment durations for buccal fixed orthodontic treatment have been reported to range from 20.7 ± 4.9 [14] to 23.5 months [5] for self-ligating brackets, and from 18.1 ± 5.3 [14] to 23.5 ± 4.7 months [15] for conventionally ligated brackets. While some authors have reported significantly reduced treatment times for self-ligating brackets compared to conventional appliances [4], with a mean reduction of up to 4 months in treatment time (from 23.5 to 19.4 months; [5]), a majority of authors [6, 7, 21] have reported that self-ligating brackets are no more efficient than conventional ligated brackets in terms of tooth alignment, or that they are even inferior [14].

Overall, mean treatment duration in our lingual multi-bracket sample was, with a mean value of 20.9 months for all groups of malocclusions and both appliances, at the same level as with buccal appliances.

### Subject allocation to treatment complexity groups

Differences in treatment time detected by different studies are likely to be due to different selection criteria for the respective samples, rather than the presence or absence of self-ligating appliances. Comparable to our study exclusion criteria, most investigators reporting on treatment duration excluded all of those cases that were classified in our study under severity degree S2, such as impacted teeth, multiple agenesis, need for orthognathic surgery, use of auxilliary fixed class II appliances, missing teeth before treatment, or cases treated with surgery [14, 15]. Parrish et al. [16] quantified the delay in de-bonding caused by separate factors of the discrepancy index and it was found to increase, on average, by approximately 6.5 months for tooth transposition, and approximately 1 month for correction of crowding, over-jet, or overbite, while Skidmore et al. reported that extractions resulted, on average, in an additional 3.3 months of treatment, or even caused a delay in de-bracketing of 5.9 months, when implemented midway through treatment [15].

### Comparison of the two lingual appliances

To the best of our knowledge, comparisons of treatment times, based on large sample sizes, for different lingual appliances have not been available. While treatment times for mild to moderate cases with the Incognito appliance is comparable to those reported for buccal appliances (22.14 mo), the WIN appliance even underscores this standard time period of about four months, on average (17.96 mo, Table 2). Even with separate consideration of severe (S2) cases, treatment durations with the WIN appliance still remain in the range of treatment times considered to be normal for mild to moderate cases treated with conventional labial-side appliances (mean: 20.5 mo; [5, 14]), while subjects with the same S2-treatment complexity that were treated with Incognito had a distinctively prolonged treatment duration, with a mean 30.62 months.

Distribution of severity grades S1 and S2 were significantly unequal within the two appliance groups (Table 4). However, the results of the ANOVA clearly show not only a significant influence of the severity of malocclusion, but also, separately and independent of the factor of treatment complexity, in relation to the type of appliance (Table 5). That is, treatment duration is, indeed, significantly different between and determined by appliance type Incognito and WIN, both in the S1 and S2 sub-groups.

Therefore, the null-hypothesis of no significant difference in active orthodontic treatment duration between either of two customized lingual orthodontic appliances (Incognito, 3 M-Unitek; WIN, DW LingualSystems) was rejected, as we found highly significant differences between treatment times for WIN and Incognito appliances with both groups of severity of malocclusion, S1 and S2.

### Lingual multi-bracket treatment as a strategy for reducing side-effects

Prolongation of multi-bracket treatment is accompanied by a significantly higher risk of developing WSLs and root resorptions [1–3]. Previous research provides hints of a decreased incidence of WSL formation in subjects treated with lingual multi-bracket appliances when compared to labial fixed treatment [8]. In terms of prevention of those side-effects associated with longer treatment durations, the WIN appliance would clearly be the better choice, especially in cases of elevated malocclusion severity or treatment complexity. The reasons for the better performance of the WIN appliance may be various and potentially include a more realistic definition of the individual treatment goal by the individual set-up, as well as an improved 3-dimensional control [22], due to the different manufacturing process and the different materials used, both leading to fewer finishing bends. Also, the improvement of some clinical protocols particularly in the phase of levelling and aligning might have contributed to the significant differences in all-over treatment time.

### Bracket failures

Based on multiple regression analyses carried out by Robb et al., frequencies of non-kept appointments and appliance repairs may explain 46% of the variability in orthodontic treatment duration and 24% of the variability in treatment effectiveness [18]. Although our study detected no statistical significant differences in terms of frequencies of bracket losses between the two types of lingual appliances investigated, the proportion of bracket failures was higher in subjects treated with the Incognito appliance (Table 1). A reason may be seen in a different extension of the bracket bases. It is suggested that appliance repairs may have been to some degree also of influence on treatment durations assessed by this trial.

### Trial limitation

This trial had a fixed time frame determined in advance. All trial patients were treated in one orthodontic center (Bad Essen, Germany) under the supervision of the same operator (DW) with either one of two completely customized lingual appliance systems that were bonded and de-bonded within the assessment period April 1^st^ 2010 – Nov 30, 2013. In this orthodontic center, all bondings were carried out with the Incognito appliance until 2/11 (bonding period 11 months), and, with the latest Incognito de-bonding in 6/13, the observation period was minimally 29 and maximally 44 months. For WIN, the bonding period was 2/11-6/12 (16 months), and the observational period for WIN cases was minimally 17 and a maximally 33 months. However, despite the shorter observation time for WIN cases due to the fixed trial time frame, there were abundant numbers of trial cases (n_Incognito_ = 220; n_WIN_ = 156), and the results of the ANOVA clearly confirmed the better performance of the WIN appliance, not only for treatments judged to be of moderate complexity, but also in the ‘severe’ malocclusion group, with adequate numbers of trial cases. Therefore, the length of the observation intervals implemented here are considered to be sufficient.

This trial did not consider the number of missed appointments, which may have a potential influence on treatment durations [12]. However, as this is a single-center trial with a total number of 376 trial subjects, it is considered unlikely that there was a significant bias in missed appointments in either of the appliance groups.

## Conclusions

Based on the findings of this study, the following conclusions can be drawn:

- Treatment duration is significantly different between the appliance types Incognito and WIN, both in cases judged to be of mild to moderate (S1), or severe (S2) treatment complexity.
- In terms of treatment duration, the WIN appliance performs significantly better than does the Incognito appliance.
- Therefore, subjects treated with the WIN appliance are expected to be exposed to lower risks of typical side effects associated with longer multi-bracket treatment durations, such as root resorption and enamel decalcifications.
